# Show Me, Tell Me: An Investigation Into Learning Processes Within Skateboarding as an Informal Coaching Environment

**DOI:** 10.3389/fpsyg.2022.812068

**Published:** 2022-03-10

**Authors:** Rosie Collins, Dave Collins, Howie J. Carson

**Affiliations:** ^1^Department of Sport, Health Sciences and Social Work, Oxford Brookes University, Oxford, United Kingdom; ^2^Grey Matters Performance Ltd., London, United Kingdom; ^3^Human Performance Science Research Group, Institute for Sport, Physical Education and Health Sciences, The University of Edinburgh, Edinburgh, United Kingdom

**Keywords:** cognitive psychology, demonstration, imagery, psychological skills, understanding, motor skills

## Abstract

Coach education is a learner-centred process, which often fails to consider the preferences of the consumer. Historically, research into performers’ experiences of coaching have been influenced by the social constructivism of learning: in short, an expressed preference for what the performer has experienced as determined by their coach, rather than their own personal preferences. Therefore, this research used skateboarding as a natural laboratory in order to explore the current practices and preferences of performers in a coach-free environment. Ninety-one skateboarders from parks in the United Kingdom and New Zealand offered information relating to their current learning practices, how they learnt about learning, and how the top-level performers in their environment were differentiated. Findings suggest that a number of learning tools are used by performers, which are closely aligned with a more traditional, cognitive view of coaching (e.g., demonstration, drills, and error usage). Results also suggest that performers deployed a number of cognitive skills (e.g., imagery, analogy, and understanding) to enhance storage of a movement as an internal representation. Finally, in the absence of formal coaching, performers use their knowledge of learning to appoint informal leaders. Implications for practice are discussed.

## Introduction

For decades, sport researchers have focussed on understanding the processes, tools and frameworks for, and of, optimum coaching practices (Côté and Gilbert, 2009). This information has then been disseminated through a wealth of channels, from the formal and peer-reviewed, to the informal and personally speculative (cf. Stoszkowski et al., 2020). Consequently, what we would typically call “coach education,” or coach development, has become a serious business. The evolution of coach development has seen coaches at the centre of the process, sometimes seeking to understand the nature of preferred support (i.e., informal or formal; Mallett et al., 2009), sometimes considering the evaluation of coaches (i.e., coach behaviours; Cushion, 2010) and sometimes even promoting the switch toward a *learner*-centred process (Paquette and Trudel, 2018). Indeed, recent research has started to consider what coaches need from the consumer (athlete; e.g., Becker, 2009), why athletes follow coaches (e.g., Rylander, 2015) and who coach development policy should be cascaded to (Dempsey et al., 2021). From this research, it is clear that coach education has proven to be impactful, influencing both the initial and continual training of professional coaches.

Whilst this investment has undoubtedly improved the provision and services of coaching, it does seem as though there is something lacking. Similarly to the situation some time ago within the education sector (Trigwell and Prosser, 1991), there has been considerably less research to address what the consumers of the coaching think constitutes the highest quality of learning experience. Indeed, the notable swing in education has seen a movement away from exploring the quality of *teaching*, instead to look at the quality of *learning* (Plowright, 2007). For example, common practice is to speak with, or canvas, pupils to explore their experiences and understand their preferences for learning (Hobson and Talbot, 2001). Indeed, this has even resulted in the development of a particular measuring tool (Evaluation of Teaching Competencies Scale; Catano and Harvey, 2011) that is built on consumer perceptions. Inspired by this advancement through a change in perspective, sport coaching may equally benefit by employing such an approach.

However, when asking athletes directly about what they prefer, a key problem relates to their socially constructed knowledge of coaching (Potrac et al., 2000; Shoukry and Cox, 2018). For example, if a performer likes the coach, it is probable that they will adhere to and endorse the methods they use. Conversely, if they dislike the coach, they will not respond so positively. Whilst this might be indicative of a thought through and considered reflection on the coaching process, it might be confounded by other much less “rationalised” perspectives. Moreover, it has been suggested that tradition and historical precedence often guide coaching practice (Williams and Hodges, 2005), which might act to limit coach education’s ability to innovate based on a performer’s needs and the learning context. In order to overcome this problem, research should look to explore the “bottom-up” preferences and behaviours of performers who have been conspicuously absent from these influences (i.e., a coach free environment) in order to verify what seems to be valued, enacted and advantageous.

Historically known for its accessibility as a sport (i.e., low equipment costs and few precursors required to be successful; SkateboardGB, 2020), skateboarding has recently become considerably more mainstream since its inclusion in the Tokyo 2020 Olympics. Skateboarding now joins the likes of freeskiing and freestyle snowboarding as a young sport in the mainstream, something which is certainly not without its challenges. Willmott and Collins (2017) and Collins et al. (2018) highlight these difficulties by suggesting that many coaches within the environment, formal or otherwise, are often left floundering, either copying the pathway of successful athletes in other sports or “overly influenced by the waves of new but unspecific sport science support now available” (Willmott and Collins, 2017, p. 2). Of most relevance, however, is that as with many other action sports, skateboarding presently remains largely coach-free (see Ellmer et al., 2020). In other words, training practices within the sport are most likely to exist because they are shared amongst, and learnt from, peers, or because it simply works for an individual. Notably, not because a coach said they should. Therefore, skateboarding, when examined outside of (new) competitive contexts (cf. Collins and Carson, 2021), offers an opportunity to explore a relatively “pure” learner-governed perspective on what works, albeit that this opportunity must be exploited quickly before the sport moves to the mainstream of coach-led activity.

To exploit this opportunity, this study aimed to explore the self-reported nature of learning and development in an informally coached, or indeed coaching-free environment, to better understand which tools were used, how performers developed and how these tools were deployed. Furthermore, if there was a difference between the top performers’ (i.e., “top-enders”) approaches compared with the developmental or simply “less proficient” performers. In exploring this, we aimed to obtain information pertaining to the focus of the performers during skill development and execution. Therefore, the objectives were as follows:

1. To explore how skateboarders learn new skills in the absence of formal coaching.
2. To establish how, and from where, skateboarders gain coaching insights.
3. To identify how and/or why “top-enders” were more successful performers.

## Materials and Methods

### Participants

Following approval from the researchers’ University Ethics Committee at University of Central Lancashire, where DC and RC were based before the beginning of the study, 102 performers were approached across seven skate parks in the United Kingdom and New Zealand. All appeared to fit the age criteria (16 years or older) and were confirmed by a gatekeeper (more details on the gatekeepers role are outlined below) for that site as a regular attendee (i.e., recognised by the gatekeeper as a frequent skateboarding participant, consistently attending each week). Of these, eight were younger than the target age and three declined to participate, resulting in a final sample size of 91 participants (9 “top-enders”, 82 developmental; 82 males, 9 females; *M*_*age*_ = 17.3 years, *SD* = 1.1; *M*_*years training*_ = 4.2 years, *SD* = 1.8; *M*_*park visits*_ = 3.1/week, *SD* = 1.2, *M*_*session duration*_ = 78 min, *SD* = 18). This “by eye then check” sampling method (Gyure et al., 2014) resulted in approaches to around 65% of those in the park at the time of visit. In other words, even though the age stipulation prevented the research team from questioning approximately one third of the available participants, the sample still generated representative results. This perception was confirmed by the gatekeepers as “external verifiers.” Using the dimensions proposed by Collins and Carson (2021), participation at each site was in a manufactured environment and regulated as a community of practice. All participants gave their informed consent to partake in the research.

### Instrumentation

As indicated earlier, the scope of enquiry was considerably broad, addressing the tasks of skill acquisition, refinement and practice. As such, it was imperative to maximise the impact for this specific group of performers, whilst also offering a useful perspective for the more general coach development literature. Accordingly, major issues which could be addressed effectively within the constraints of the study environment were considered first. Purposefully, we sought to collect these data atheoretically in an attempt to maintain a lack of bias toward any literature-informed theory or framework of formalised coaching. This led to the development of a draft set of questions that was initially piloted with six performers from two skate parks not involved in the main study. A process of cognitive interviewing (a tool to administer open-ended questions in an effort to review question understanding and nature of response, e.g., “did you find any of the questions difficult to understand?”, “what do you think this question refers to?”, “could any of the questions be rephrased to help you understand them more?”; Beatty and Willis, 2007) followed this pilot process and resulted in three changes that offered greater clarity against issues raised. The final interview tool was comprised of:

1. Consider difficult tricks or sequences you learnt recently or are learning.
  (a) How are you learning/did you learn them?
  (b) What did you use to help?
  (c) What else would have helped you?
  (d) What do you do if/when you make a mistake?
2. Where have you picked up ideas on how to get better?
3. Who is/are the best performers in this park? (used to identify the “top-end” performers whom were approached after being identified).

Based on this line of questioning, participants were free to interpret and express their learning experiences as relevant to them and unguided toward any theoretical position.

### Procedure

A member of the research team originally approached the management of each skate park to seek permission to undertake this research and approach performers. This approach was made in association with a park-specific and previously identified gatekeeper who had been recruited through personal contact. Gatekeepers were uniformly over 21 years old and experienced riders themselves. Most importantly, they were regular attendees at that particular skate park and were well known to the other performers at that venue.

Following approval from skate park management, one member of the research team (two researchers collected data across the countries) attended the park with the gatekeeper, approaching individuals together, to invite them to take part. Individuals were only approached if they were recognised by the respective gatekeeper as being regulars at that particular park. A key and early part of this approach was an explanation of our purposes, provision by the researcher of photo identification and an explanation as to how the study would work from an ethical perspective. In brief, participants were guaranteed anonymity. Indeed, the research team deliberately did not record their names, but only took age and participation data for the purposes of describing the sample. Each participant was assigned a number at this stage to enable future withdrawal upon request.

Since the study aimed to obtain participants’ views on the topics addressed in the questions, no *post hoc* interpretative analysis was intended. Rather, accuracy of recording and individually confirmed viewpoints were sought at the time of interviewing. Accordingly, questions were asked by the investigator whenever the participant’s statement was unclear or could be misconstrued. Importantly, however, probes were used sparingly to avoid any tendency to lead the participant. For example, to avoid leading participants into giving the researchers the answer they thought we might be after, and any potential researcher-bias based on formal coaching literature. Probes were only utilised to seek clarity, such as when using sport-specific language, or to check the researchers’ understanding of the participants’ statements. This approach resulted in a conversation, with the interviewer reporting back what had been heard and asking for the participant’s confirmation whenever things were not clear. This process received further clarification by the gatekeeper, especially when technical skateboarding terms were used. This process was our best attempt to avoid any issues caused by the lack of member reflections when addressing the trustworthiness of our research (see section “Trustworthiness” below for additional details and steps taken).

Interviews lasted between 22 and 40 min (*M* = 33.3 min, *SD* = 6.9), with a roughly equal split of participants contributing across the four data collection sites. At each site, every individual identified by the gatekeeper as meeting the participant criteria was invited to participate. Upon completion of the fourth and final site visit, data processing commenced with the view to establish levels of data saturation.

On completion of each interview, the researcher handed the participant an information sheet, with their participant number noted. This provided written details which had already been explained to the participant, inviting them to reflect on the participatory conditions themselves and, if under 18 years old, check these with their parents or guardians at the earliest opportunity. On this sheet, the lead researcher invited phone or email contact if either participant or parent/guardian did not wish them or their data to be included in the study. Importantly, no such calls were received although we did receive 10 inquiries about the study with interest in the results. Importantly, this information sheet also provided details of the University complaints procedure in case parents/guardians or participants had concerns about the process. Once again, no such calls were received.

### Data Analysis

To some extent, these data can be considered as inductively analysed because the researchers held no expectations or structures (skateboarding specific knowledge) prior to the investigation. Against the first, and arguably overarching objective, a more thorough exploration was warranted to tease out any nuances across participant responses. Reflecting qualitative innovations by Braun et al. (2016) and Braun and Clarke (2019), raw data codes were compiled in order to identify Central Organising Concepts (COCs; Braun et al., 2018). This was a comparatively straightforward process since responses had already been clarified/confirmed by participants. Reflecting the pragmatic nature of this research, utilising reflexive thematic analysis allowed the data analysis process to accurately reflect the participants’ experiences and preferences for learning in an inductive manner (Braun et al., 2018), as the typical “checking” processes had been completed at the point of data collection (Denscombe, 2007). Due to the simplistic nature of Objective 2, the findings were reported by grouping the same responses from participants, as verified at the time of interviewing.

Moving forward, having established the key data themes, Objective 3 was answered by comparing data which were already analysed between “top-enders” and the remaining participants, using a more deductive (against the established COCs) thematic discourse analysis (Clarke and Braun, 2014). This objective was an extension of the first (i.e., how might “top-enders” differ in learning), therefore, pragmatically this more fluid and dynamic analysis tool was deemed pragmatically appropriate.

### Trustworthiness

In addition to the steps outlined above, we sought to ensure maximal trustworthiness of these data in order to support the pragmatic philosophy underpinning this research. We were especially aware that researchers are not able to extract themselves from their own experiences, and therefore biases (Denzin, 2017). Accordingly, interactions were almost entirely participant driven, with the investigator completing “real-time” member reflection by reporting back responses to each participant. As stated above, the comparative simplicity and straightforward nature of these responses were major factors in deciding on this approach.

Responses were also subjected to two “external” checks. Firstly, a digest of the data was shared with each gatekeeper, asking for their opinions as to the veracity of the data. In short, whether anything that they had heard, or that the researchers reported back to them, sounded odd or out of the ordinary. No such opinions were expressed, with gatekeepers “endorsing” the results as representative of their own experiences, knowledge and actions in skateboarding. As a further and final check, the results were shared with two experienced international action sport coaches (one from the United Kingdom and one from New Zealand, both with over 15 years’ experience as full time coaches) who were asked the same questions; that is, whether anything struck them as surprising or different to their experience, together with their observations of the messages within the data. Although not skateboarding coaches (one was a free skier whilst the other coached snowboarding), both were very in touch with the action sports scene and familiar with skateboarding through their work with their own performers. Once again, the results were endorsed as presenting a true and accurate picture of the milieu by both subject matter experts (SMEs). One of these SMEs, Sean Thompson, the Head Snowboard Coach for New Zealand, offered the following insight:

Being a lifelong action sports enthusiast, I have dedicated decades of time both learning and coaching board sports such as surfing, skateboarding and snowboarding. My current role as the Olympic Slopestyle and Big Air snowboard coach puts me in the frontline of working closely with an array of athletes in a similar demographic to that studied in this paper. All findings and correspondence from the riders within the paper are what I would expect to be the norm from that age group in that sport.

Both coaches were happy for their names to be reported. The other was Pat Sharples, Head Coach of Snowsports GB.

Finally, the data analysis approach was informed by the research team’s applied experiences, one of which was not involved in the data collection process (37, 13, and 8 years’ experience in sport coaching, supporting performers up to international level). In contrast, and positively, the lead researcher had little understanding of skateboarding participation, without explicit expertise which could bias their view. The team also brought considerable literature-derived knowledge or theoretical knowingness (Braun et al., 2016). Notably, this allowed the data analysis process to accurately reflect the participants’ experiences and therefore provide practical information surrounding a practical problem (Denscombe, 2007), whilst also offering sufficient background to understand and interpret their perspectives (which, again, were confirmed through the “checking” process at the point of interview). As well as the various checks reported so far, the third researcher with expertise in coaching theory and practice acted as a critical friend. Specifically, this knowingness reflected the mechanistic principles outlined by a variety of theoretical approaches. In this way, data were meaningfully analysed through reflexive, transparent engagement, thus working toward a “richer more nuanced reading of the data” (Braun and Clarke, 2019, p. 594).

## Results and Brief Discussion

Results with brief discussion points are presented in three sections to reflect the research objectives. Against the first research objective, a summary table is provided to offer an overview of data (a percentage respondent score is included to illustrate how often the COCs were mentioned to represent commonality, as opposed to signifying their importance; Taylor et al., 2017) followed by an exploration of the COCs (in some cases, for ease, presented together). Results for Objective 2 are reported as an overview of participant responses. Finally, Objective 3 is presented as a derivative of the first two objectives, by highlighting the distinguishing characteristics of “top-enders.”

### Objective 1: How They Learnt

Reported learning methods are summarised in Table 1, with exemplar quotes used to provide detail for each theme.

**TABLE 1:** Participants reported use of learning tools.

Central organising concept	Reported by	Exemplar quotes
Analogy, feel and internal representations	44 (48%)	To help me get the rhythm I’ll often see a picture in my head that makes me feel like I want it to look. For example, lots of the time. I’m seeing myself surfing a wave. I might see someone interviewed on [skateboarding website]. He will be talking about something else he’s done that helps him get the move right. “Whipping cream” when riding a bowl is one that’s helped me a lot.
Attention	78 (85%)	Lots of time I’ll pay attention to what I look like. After all that’s a big motivation for being here. Every so often I’ll work on what the move feels like. I’ll stay inside my head and get the feel before I do it.
Imagery/Mental practice	85 (93%)	I’ll lie awake in bed running through a trick – what it will feel like and, to be honest, how good I’ll look! When I get the chance to watch someone doing a target trick, I’ll watch then try and feel how it would be for me. I’ll do that loads of times till I think I’ve got the idea.
Demonstration	80 (88%)	I always take the chance to watch someone perform. I learn so much from it… I look’ specially when the good guys are riding, I’ll take a sneaky peek!
Explanation	78 (85%)	I find it really useful to talk things through with other riders. They often highlight things I haven’t thought of. I love it when someone agrees to talk me through how they’re doing something.
Error usage	45 (49%)	I’ll watch a run several times. I want to see what I’m doing wrong so I can correct it. I like to talk over mistakes with my mates, I want to see what they think I should do.
Practice	90 (99%)	This is all about practice…repetitions till I look smooth and effortless. My aim in practice is to look consistent and smooth…I want to flow.
Planning and preparation	80 (88%)	I usually think about what I will do on the way to the park…set myself some challenges or whether I’ll just ride depending on how I feel. I take a competition schedule and work out what I need, when I need it.

#### Analogy, Feel, and Internal Representations

When practicing or learning skateboarding skills, participants reported a high prevalence of explicit and analogy learning strategies. Specifically, these explicit and analogy strategies are conscious patterns of thought, or foci of attention, that help to generate the movement mechanics to be performed (Poolton and Zachry, 2007). Analogies were reported particularly often, interestingly in the form of pictures and thoughts of surfing. As expressed by this rider, “I love to ride round a bowl and picture myself cutting up and down a wave,” or from this experienced rider: “to keep my balance I will often imagine a piece of string pulling up from the top of my head.” As such, reported thoughts about movement patterns were directly and positively related to the completion of the task, skill or “trick.”

Another interesting finding was the deep understanding of tricks or sequences which many participants found really important for their learning, such as “I don’t only want to know what it looks like or what it feels like when I do it well. I want to understand how it works from a kick flick upward.” Another more experienced 19 year-old rider explained:

I guess as the older dude around the park I get a lot of young guys asking my advice. I always want to make suggestions to them that develop their understanding of what they’re trying to achieve. I use words, symbols, stories [probing suggested this to be metaphors] or pictures to do this.

Accordingly, participants reported the development of an internal, or mental, representation as defined by hierarchically stored movement-relevant knowledge (Schack and Mechsner, 2006) to support their own learning and that of others. Notably, however, these were often driven by a mixture of internal and external constructs, for example: “I really want to know how a sequence will run before I do it. I’ll store and practice that usually as a combination… imagining it and what it looked like against the ‘list’ of moves,” “I run through a list of moves in my head and the rhythm…often I’ll get the rhythm of the moves from a favourite piece of music. You mentioned ‘Eat, Sleep, Rave, Repeat.’ I use it!”, or from this 17-year-old: “I’ve actually set up a run list at home with video cuts for each move. I’ve been using that to put together an ideal run or sequence…putting things together as I can physically do them.”

#### Attention and Imagery/Mental Practice

Interestingly, there were clearly a number of participants who thought about what they attended to, when and why. External focus was commonly used (often facilitated by use of video) for example, “I’m worried about what I look like doing the run, how smooth it looks and what impression it’s going to make.” There were notable situations, however, in which participants also reported using an internal focus. “As I’m watching someone do a trick, I’m trying to imagine how that will feel…I watch out, then think in.” Or this 18-year-old: “I often run through the rhythm and feel of the sequence just before I do it to get me ready.”

Unsurprisingly, use of imagery was a prevalent tool used by participants. Around 90% of participants reported using imagery in some shape or form, although two broad categories were apparent (Cumming and Williams, 2013). Firstly, mental run-throughs at home or away from the park venue. Content seemed to include elements of mental rehearsal and “ideal performance” motivation; sometimes in combination. For example, one participant recalled:

When I first went for a “Crooked Grind” [a slide along a rail on the front of the board] I fell and broke my nose. After that, I would watch a demo video on [website], seeing myself do the trick, then feeling how it would be if that were me.

The second category related to imagery *at* the park, which was reported as both preparatory (mental rehearsal) and as a combination with action observation (see section “Demonstrations and Explanations”). For example, as this participant reported, “So when I was working on improving my Nollie Flip [jump up as board rotates under you then land] I would watch a video on my phone, then run through how it would feel. So watch, feel, then do.” This combination of mental run-throughs in combination with some form of “instruction” (either watching video, receiving instruction or watching someone else) was extremely common.

Interestingly there was some evidence for a switching of attention, often in a “whole-part-whole” approach. For example, “I always find it important to think through the whole run and what it looks like before going inside my head to check the feel of the difficult dismount or bit in the middle.” Or this 16-year-old: “what we’ve been talking about, inside my head or watching myself or focussing on what the thing will look like; I use them all…it depends!” In summary, a mix of external and internal foci were apparent in this sample (Oliver et al., 2021).

#### Demonstrations and Explanations

Across participants, demonstrations played a big role. Almost all used others as formal (show me how) or informal (covert watching) models. Additionally, although not strictly explanations, verbal input from other riders was extremely common across our sample, for example something Hollett (2019) has termed “vibing,” was a common feature. This involved small symbiotic relationships across riders. These “mutual interest groupings” or communities of practice (Culver and Trudel, 2008) then used video and still images, usually from phone cameras or similar, as the basis for after-action debriefs on what had happened and to identify areas for improvement. As one rider put it, “yeah, it’s really important to get a perspective from my mate on how I’ve done,” and another, “we’ll usually work in the evenings, usually on social media especially at the moment, debrief on progress and set some targets for what I need to change.”

Loss of credibility seemed to be the only barrier to using demonstrations as an overt strategy, as explained by this participant: “^****^ it wouldn’t be cool if I was walking round staring at all the other skaters!”. Subsequent to watching, either overtly or covertly, participants would try to work out what they would have to do to accomplish what they had seen. In this form, demonstrations were used in a juxtaposed fashion through combinations of imagery and observational learning. Examples from participants include: “I’ll pick a star performer and watch how he does a sequence then go and try it myself, trying to reproduce what I saw with what I’ll feel,” or:

I’ll often ask for advice or if someone minds me hanging with him. Often, I’ll approach them and say “hey that was sick…how do you do that” and they’ll usually show me and offer a quick talk through. I find I learnt an awful lot from listening but don’t tell my Mum!

It was interesting that, in the absence of formally appointed or employed coaches, our participants established surrogate coaches through peer learning and teaching. Even more interesting was the extent to which, although they should be termed informal, the impact of these relationships were so powerful as to give them an almost formal feel. In fact, participants with experience of other sports drew this analogy themselves, for example “I would probably pay as much attention…hey, perhaps even more, to my friends at the skate park as I would to the stuff I get from my football coach.” Alternatively, this participant highlighted “I’ve had a lot of coaches in the activities I’ve done up to now. I have to say that working with my friends is far more effective because they have a real understanding and feel for what we’re doing” (Fransen et al., 2015).

#### Error Usage

Getting data on the use of this tool was notable in that almost all participants provided lots of information but, almost always, only after probing. Several spoke of the need to be accepting of errors, such as this rider: “You’re never gonna be any ^****^ing good at this if you don’t have lots of ^****^ ups” or this,

You’ve got to accept that you’re going to take more than a few falls…it isn’t great in front of your mates but to be honest the hardcore boys in here just accept it and even encourage you to have another go.

One big feature of the groups’ learning strategies described below, was how participants used their peers, together with video feedback, to help them correct errors (Guadagnoli and Lee, 2004). For example, “My mates are great. They notice differences or problems, point them out and suggest changes,” “If I do a run, especially if I’m trying for something in competition, I rely on my mates to help me look at the run [critically] and work out where I can make improvements” or finally from another participant:

I think it’s crucial to use your ^****^ ups positively. I want to work out what I’ve done wrong and how to correct it. To do that, I use as many different inputs as I can…teammates, video, how it felt, the whole lot.

Error correction and the tools to do it were seen as particularly important for competition (cf. Poolton et al., 2005), as shown by this participant quote:

I might be in something at the park where I’ve got the best of three runs. If I land the first one that’s great. If I ^****^ up, I need my mates and the video to help me get it right next time.

#### Practice

Unsurprisingly, practice was mentioned by almost every participant. Unsurprising because, for many, practising and refining their skills represented the whole joy of the activity in this aesthetically driven sport. Drilling, repeating moves over and over again, was a major feature. “I have to get my moves straight. I keep going and going ‘til I just know I can do that move wherever I am.”’ Or this 16-year-old who seemed to be using a form of overlearning: “I have to have the basics…I have to be able to ollie [a jump up or on to a feature with the board] wherever I am.” Interestingly, this desire for skill transfer did mean that participants would try out the same skills in a number of different sites, either within the same park or on trips to others. Importantly, however, especially against ideas like “repetition without repetition” (Bernstein, 1967), they would usually get this mastered in one situation before trying it elsewhere. “When I started, I hammered the stance-push-stop basics at home. Only then did I feel comfortable to go out to the park…to ride in public!”

Participants reported several different features common in other skill acquisition scenarios and also seemed to draw on ideas from other action sports. For example, as previously highlighted, whole-part-whole seemed important for those getting a sequence of moves down (Hanin et al., 2002). “I’ll plan a run across the park then use that as the base for practice. I might do the whole run, then work the rail in the middle, then put it together and then go again.” At a higher, session level, performers were very aware of setting up a theme or target for the day; some in advance but some in a more *ad hoc* fashion (see section “Programming and Planning” below). Interestingly the idea of push-drill-play, recently discussed in free skiing and snowboarding (Collins et al., 2018), seemed to resonate with participants even though they had seemingly never heard of the original idea. “Some days I’ll get to the park and it’s having it…I’m there on a mission. Other times I’ll just go hammer one or two moves. Other times I’m just going to ^****^ about with the guys.”

Finally, as a small but distinct subcategory, there were several participants who just preferred to go on their own. These “solo performers” seemed to understand the sense in their peers using others, but it was just their personal preference to practice alone. For example, one 18 years old states:

I’ve never been one for the crowd, especially when I’m putting new stuff together. Even when I started, however, I’d much rather go away on my own and get things sorted. It was almost like people being around were a distraction…or a challenge to what I was trying to achieve.

#### Programming and Planning

We have already mentioned participants’ habits about making decisions on what they would do at each visit. Clearly, and in the absence of any formal designated coach, no written structures were apparent. Interestingly, however, participants themselves imposed structures mostly at micro or session level, as well as a meso (monthly) and macro (yearly) level (Bompa, 1983). From a micro perspective we would reiterate that, with certain exceptions, riders would usually arrive at the park with a predetermined plan; albeit that this might have been arranged on the bus journey to the park. One participant stated “I don’t just like to turn up. Course it ain’t like school but I want to know what I’m gonna get from being there, what I’m gonna do, even who I’m going to meet.”

At the meso level, many participants used both vibing and prior discussion to develop at least plans of intent; an outline of what they wanted to achieve over the next few weeks. “I watch a lot of video and visit a lot of skateboarding websites and that gets me interested. It gets my juices flowing about what I want to try and achieve next.” Or this 16 year-old: “I watch videos and websites but that’s the sort of an external pressure of course. I also want to keep up with the leaders at [name of park].”

Macro level planning seemed to be apparent only in a minority of participants with a regular competition schedule or the view of getting involved in competing. “I know what comps I’m going for…it determines where I am, when and what I’m doing.” Or this 18-year-old:

I’ve really got into competing at skateboarding. I’d say that has taken over as my main motivation. I want to do well…I want to establish a reputation for myself and start getting some of my videos on Instagram or YouTube. I can see a genuine career in this.

### Objective 1: Brief Discussion

Based on the analysis above, it is clear that participants use a variety of tools and skills to develop their ability in skateboarding. Perhaps most revealing from these data was a clear emphasis on cognitively oriented structures and processes. For example, the use of mental imagery was utilised from different perspectives, and for different purposes. Most notably, however, participants expressed that the “thinking through” of skills was a common feature of their initial understanding and the movements’ continued execution. In this way, knowledge was considered to underpin progress, both in terms of the skill itself and as the rider’s ability developed. As such, these data tend to support the development of individualised movement representations that provide a scaffold for interpreting information within the skateboarding environment, and for guiding the skill execution.

These explanations are well-aligned to the multi-level framework proposed by Schack et al. (2014). Data suggest that individual (lists of moves) and/or clustered (combinations of moves) elements of stored movement representations (Basic Action Components; i.e., knowledge of what to do) need to be integrated with coherent sensory representations of what the skill should “look like” (i.e., perceptual effect-representations) and mental control strategies (e.g., pre-performance routines) to provide a most elaborate/complete understanding of technique development (see Schack and Bar-Eli, 2007; Schack et al., 2014). Consequently, this framework would support the sensible use of both internal and external foci by performers to be able to fully understand and perform skills (Collins et al., 2016).

Despite this cognitive emphasis, data support a growing realisation within sport science and coaching research for an interactive understanding of processes and practices. In this particular case, the cognitive elements were expressly influenced by social factors. Importantly, who was observed within the park, the frequency of overt watching and the impression presented to others (i.e., the aesthetic of the skill) constrained the utility of mental skills. In summary, how riders learnt was reportedly grounded in cognitive mechanisms that were influenced by multiple environmental considerations.

### Objective 2: Where They Learnt About Learning

As stated earlier, our interest in this particular participant group was the almost complete absence of formally appointed or explicitly recognised coaches. As the sections above demonstrate, however, there was clearly coaching in place and this process was both acknowledged and valued by our participants. Once we had explored early responses about how to get better, which initially were mostly related to technical aspects, we then managed to focus on why participants were practising in the way they were and where this might have come from.

There were many responses which fell into the tacit category (Nyberg, 2014). For example, this 16-year-old: “It felt comfortable watching and copying…I feel like I have done that my whole life.” For these sorts of responses, participants seemed unaware of where the techniques had come from or unable to offer any rationale as to their use. Answers of the “it just does [work], so I use it” category were the most common with 58 participants (64%) responding in this way.

In addition to these, however, there were a number of perhaps more thoughtful participants who offered a greater depth of response. For many of those participants, ideas and approaches were transferred from their experiences of skill learning and practice in other environments. For example: “I guess I just think about the way we do it at school. It makes sense so I use it in the park.” Or from this 16-year-old: “I used to go to both gymnastics and judo clubs and I guess how I practice here has been quite influenced by the stuff we did there.” We obtained similar responses from 17 participants (19%).

Other participants reported gleaning techniques from websites, mostly in skateboarding but also notably in other similar action sports (Jones, 2011). “I’ve watched several videos on [skateboarding site] which have interviewed top riders. They all talked about imagery or visualisation as a technique. I tried it and it works.” Or from a 16-year-old: “I’ve seen even the stars trying and failing a number of times, looks like they go away and hammer the practice, if it’s good for them it’ll work for me.” Websites were mentioned by 16 from this sample (17%).

Finally, a small number of participants had actually sought out help from books, social media and websites specifically on the pedagogic principles. “I got this book for Christmas that talked about coaching and pretty much that became my Bible.” Or “I get great ideas from social media sites and blogs on coaching…I try them and if they work, I add them to the mix.” This more “academic” approach was apparent in 12 of this sample (13%). As should be clear from the totals, some responded in more than one category.

### Objective 2: Brief Discussion

Many other action sports already operate within a formal coaching culture, albeit that those coaches have usually received training in another, more traditional sport, then transferred these skills into the new activity, supplementing it with books, internet-based knowledge and communities of practice (Collins et al., 2019). Similarly, a proportion of participants expressed a desire to develop their knowledge further, predominantly using sources of the same nature, such as websites and social media. This further highlights the importance for coaches offering information on these sources to consider both quality and bias (Stoszkowski and Collins, 2016). For example, high-level coaching must consider the age and stage of specific learners, in this case youths, and the extent to which generic online material is most appropriate for these participants. It may well be that a combination of “expert” modelling prior to practice, followed by a combination of “self,” “coping,” or “self-coping” models offer a better long-term solution; in short, it depends!

### Objective 3: Top-Enders

Finally, we were able to interview nine individuals of the 11 top-enders identified. It would be wrong to define these individuals as experts. We applied no performance criteria and their “appointment” to this status was clearly context specific and based on group perception. That said, there were several differences in the practice behaviours of these individuals which, whether causative of, or associated with, their status, seem worthy of note. Results were extremely similar to the other participants, with one or two notable exceptions. Firstly, 100% were keen and consistent consumers of external sources (social and other media) on skateboarding. “I need to look at the sites at least twice a week to stay up to speed…it’s where I get my edge,” “I want to see what others are doing – the ideas help me to improve and also direct my practice.” Original ideas were usually sourced from other environments whilst only a few were genuinely creative in focus.

As a second difference, top-enders seemed almost “error seeking” in their exploration of new alternatives (Hodges and Lohse, 2022). “If I can do it this way then why can’t I do it that way…if someone else is doing it like this then why can’t I do it like that” or “I’m always looking to do the new and peculiar especially when it comes to putting moves together.”

Finally, these participants seemed a lot more self-driven and experimental in their activity (Mallett and Hanrahan, 2004). “I tend to set myself some clear targets, but these are based on what I want to achieve… it’s all about me!”, “When I come to the park, I tend to play with purpose…to just ^****^ around to see what I can come up with.” Or this 21 years old (one of the elder statesmen) “Things have changed as I’ve got older; I used to watch the others all the time; picking out a guy or a trick that I wanted to copy; but not now.”

### Objective 3: Brief Discussion

Against this objective, the exploratory nature of these responses are similar in this regard to that of expert breakdancers, reported by Shimizu and Okada (2018). Notably, these local leaders appeared to be far more committed “students” of their sport, when compared with other participants. Evidence of information-seeking beyond their immediate domain, in order to stay ahead or provide an edge to performance, reflects present understanding of creative expertise (Mishra et al., 2015). As with the previous objectives, these participants’ account of their practice were grounded in cognitive processes, often developing a more detailed and in-depth analysis of their skills.

## General Discussion and Recommendations

The objectives of this study were to explore how skateboarders learn new skills in the absence of formal coaching and establish how and from where these skateboarders gain coaching insights. Finally, to identify how and/or why “top-enders” were more successful. Overall findings revealed that participants utilised a number of tools and aids to acquire and enhance their skills, often sourced socially (through both media and peers; Jones, 2011) and interpreted by participants through a cognitive perspective of learning (Schack et al., 2014). Following our brief discussions above, there are a number of important points of discussion that could usefully inform the coaching literature.

Firstly is the perceived theoretical underpinning of learners’ development as being largely cognitive in nature. Not only did this relate to the way in which learners practiced, but also the knowledge they acquired to help consolidate skills away from the park. Clearly, this perspective is in contrast to opposing ecological approaches (something we will come onto below), but data are also contradictory of approaches *within* the cognitive paradigm and promoted in sport coaching literature (Winkelman, 2017). Specifically, participants reported the active use of cognition to understand, develop and control their movements in pursuit of higher skill levels. Such a mechanism of learning is counter to that promoted by implicit motor learning, which suggests that learners practice under conditions to actively prevent the accrual of knowledge pertaining to the technique to avoid subsequent breakdown under pressure. For example, this approach would promote practice without errors and/or dual-task conditions to consume working memory with task-irrelevant information (Poolton et al., 2005; Gabbett and Masters, 2011). Indeed, the relevance of implicit motor learning has recently been raised as an ill-considered approach to learning complex sports skills (Bobrownicki et al., 2019) and would appear to not reflect the way in which these participants told us that they learnt. Therefore, despite being underpinned by the cognitive approach, we would suggest caution toward any recommendation of implicit methods that are currently popular within some coaching communities.

In support of active conscious control as an underpinning mechanism of learning and skill execution, training by the performers resembled the use of “contrast drills.” Previous studies have explained that the aim is to compare and contrast an existing and desired movement version by generating a new alternative and then consciously distinguishing between the two; in turn, this differentiation serves to create a realisation of the change required (Carson and Collins, 2011). Central to this process is an athlete’s understanding of the movement, before internalising the changed component to subconscious control (Carson and Collins, 2011). This could be seen, for example, during the use of errors, which participants used as tools to develop understanding by better realising what they were trying to avoid (Light and Harvey, 2015). While a large majority of research in sport coaching and motor learning has focussed on practice schedules such as blocked and random practice (e.g., Lage et al., 2015), the use of contrasts at this stage of learning is relatively, if not completely, absent and presents a beneficial development within the coaching literature.

Furthermore, the reported use of attentional control within these data was expressed as a dynamic process in both direction and purpose. Sometimes, focus was on the skill production (i.e., what the movement looked like) and then switched internally (i.e., what the movement felt like) once the performer had identified the movement(s) of interest; representing the switch from “attention” directed externally within the environment to an internal state of “intention” to retrieve the movement from memory, as seen in many target sports prior to skill execution (e.g., Hatfield et al., 1984; Loze et al., 2001). Indeed, the common use of “watch then image” before “doing” as a method is very similar to ideas suggested in karate by Smith et al. (1997) and recently examined in darts by Romano-Smith et al. (2019). The combined use of alternated observation and imagery was commonly reported as offering a means to “internalise” what was being watched (cf. Hall et al., 1998; Fournier et al., 2008). We did not probe on the modalities of this process, on the basis that the explanation of constructs would have been too theoretically leading. Notably, however, observation of several participants (watch – look away – watch – repeat) was highly suggestive of the external visual *then* internal kinaesthetic strategy suggested by Smith et al. (1997). In fact, recent research into the mirror neuron system would suggest that the extent of neural activation during this perceptual process to be enhanced with improvements in the skill execution itself (Calvo-Merino et al., 2006). In other words, as the performer’s ability to empathise with the experience of the model increases, so too does the quality of information extracted from the observation and, in turn, ability to use it for memory retrieval purposes. Overall, data from participants suggests that research should explore mental strategies as non-dichotomous constructs when seeking to understand how they can usefully benefit learning and performance (cf. Wulf, 2016; Collins, 2021).

From a physical perspective, participants reported the almost ubiquitous use of drilling, comprising of many repetitions of the same skills. Such practice is commonly associated with traditional information-processing approaches to learning (Williams and Hodges, 2005), however, the use of errors expressed by participants suggests that even in this context the repetitions were not the same and therefore would not constitute effective practice. This concept is, however, congruent with the “repetition without repetition” (Bernstein, 1967) notion synonymous with dynamical systems theory (Kelso, 1995) whereby variability in execution is deemed a positive attribute for future adaptability. Recent advances in this thinking, however, have posited that the degree of repetition across the different movement subcomponents is differentially meaningful (Scholz and Schöner, 1999). For clarity, some elements of the skill should be trained to purposefully demonstrate more consistency than others *because* they are important to achieving the desired outcome. Whether or not these, or most probably the other, movement subcomponents could be considered as being self-organised is beyond the scope of this paper. Our data suggest, at least, that participants understood their cognitive intentions when repeating a skill as contributing toward movement effectiveness of those subcomponents; that is, what the performer was trying to work on became more consistent across repetitions. Accordingly, motor skill development may be better considered as a blend of cognitive and non-cognitive processes (e.g., ecological direct perception) interacting within and across each repetition (cf. Collins et al., 2021). As such, coaches should consider *what* they ask their performers to focus on *and* the extent to which the training regime promotes variability to support the flexible execution of non-essential movement subcomponents.

Irrespective of the motor control perspective taken (cognitive or ecological), data pointed toward a clearly complex and biopsychosocial learning processes for participants. For example, considering a social perspective to what participants reported, in the absence of formal coaching, participants held in high esteem acted, with perhaps equal effect, as surrogate or peer coaches. The only barrier to seeking “formal” coaching status appeared to be social embarrassment, or perhaps ego! Indeed, social dynamics within the park also heavily impacted on the use of practice and learning tools. Jackson and Beauchamp’s (2010) work on metaperception within athlete-athlete relationships is relevant here, because it highlights the importance of understanding relationship dynamics as another key factor in response to coaching buy-in. In other words, getting better at performing the skills (Bio), is enhanced by identifying and extracting task-relevant information from a model (Psycho), which is more likely when that model is highly valued (Social).

Of course, and again reflecting the biopsychosocial nature of learning, there were individual preferences amongst participants that coaches should consider. This aligns with the suggestions from Ellmer et al.’s (2020) scoping review which highlighted the individualistic and typically self-regulated nature of learning in sports similar to skateboarding. As suggested by Nokes-Malach et al. (2015), self-identified solo learners seemed to suggest that others “got in the way” or made them “feel too busy!” These attitudes, along with several identified in the present study (e.g., feeling responsible to push themselves as an independent “top-ender”), need to be considered by coaches in offering more nuanced approaches toward performer development.

Finally, it is worth considering the further comments offered by one of the SMEs in relation to how a coach’s knowledge/understanding of participants could positively impact on potential biopsychosocial interactions. Thompson expressed:

The language used in responses from the skateboarders was of interest to me, phrases such as “I want to understand” and “I really want to know.” This got me thinking about curiosity and the role it plays within the learning process. In particular, how curiosity can drive progression and therefore the risks of coaching not nurturing ones natural level of curiosity (S. Thompson, personal communication, 28th November 2020).

It seems clear that Thompson, an experienced coach in a pursuit not dissimilar from skateboarding, expresses the importance of understanding as part of the skill acquisition and developmental process. He went on to explain that a key feature of this understanding exists due to the nature of the physical pursuit.

I see this on a daily basis working with my current athletes. The more curious an athlete is about an area of performance the more they are willing to delve into it to seek performance gains. This becomes even more apparent when the level of risk is high, especially in progressive sports like skateboarding and snowboarding. Once the curiosity is there, the “whatever it takes” mindset kicks in and the reward of landing a new trick out values the risk of injury (S. Thompson, personal communication, 28th November 2020).

### Conclusion and Implications

This study was designed to explore a modern youth phenomenon; namely, unstructured and non-directed practice in an informally/socially judged activity. The main purpose was to see how young people learnt skills in an activity when it was “coach-free.” There were clearly many different and often contrasting ideas with existing literature, however, perhaps the clearest idea to emerge is the necessity for coach decision making to be contextually driven and focussed on both the needs and preferences of the learners (cf. Vinson and Parker, 2019). Regarding the choices about, and applications of, learning strategies in these coach-free performers, participants predominantly reported cognition to underpin their learning, in addition to social factors as important to enacting these mechanisms. Of course, however, the coaching tools reported by the participants may also be suggested as representative of an ecological approach, through the use of constraints for example (e.g., FTN, 2020). In any case, a performer’s learning preferences are likely a socially constructed phenomena, and therefore all previous research which has sought to support a particular coaching approach has been influenced by this. Therefore, it seems sensible that research should look to explore in greater detail the preferences and behaviours of performers who have been conspicuously absent from these influences. Moreover, we would welcome data from a range of methodological approaches, but encourage researchers to consider the purpose of their research and whether or not it *can* relate to and directly translate to the learners/coaches involved.

## Data Availability Statement

The raw data supporting the conclusions of this article will be made available by the authors, without undue reservation.

## Ethics Statement

The studies involving human participants were reviewed and approved by the University of Central Lancashire. Written informed consent to participate in this study was provided by the participants’ legal guardian/next of kin.

## Author Contributions

RC ran the project and contributed to leading on ethics submission, data collection, data analysis, and write up. DC supported in data collection, collected all the data in the second country, and wrote the final manuscript. HC acted as a critical friend throughout the data analysis process, insured maximum rigour throughout the process, and supported significantly with the write up of the article. All authors contributed to the article and approved the submitted version.

## Conflict of Interest

Conflict of Interest: RC and DC are employed by the company Grey Matters Performance Ltd. The remaining author declares that the research was conducted in the absence of any commercial or financial relationships that could be construed as a potential conflict of interest.

## Publisher’s Note

All claims expressed in this article are solely those of the authors and do not necessarily represent those of their affiliated organizations, or those of the publisher, the editors and the reviewers. Any product that may be evaluated in this article, or claim that may be made by its manufacturer, is not guaranteed or endorsed by the publisher.
